# Piezocatalytic ammonia synthesis from seawater nitrate for sustainable nitrogen cycle

**DOI:** 10.1093/nsr/nwag008

**Published:** 2026-01-08

**Authors:** Zhiguo Niu, Yang Fu, Tianyi Ma

**Affiliations:** Centre for Atomaterials and Nanomanufacturing (CAN), School of Science, RMIT University, Australia; ARC Industrial Transformation Research Hub for Intelligent Energy Efficiency in Future Protected Cropping (E2Crop), School of Science, RMIT University, Australia; Centre for Atomaterials and Nanomanufacturing (CAN), School of Science, RMIT University, Australia; ARC Industrial Transformation Research Hub for Intelligent Energy Efficiency in Future Protected Cropping (E2Crop), School of Science, RMIT University, Australia; Centre for Atomaterials and Nanomanufacturing (CAN), School of Science, RMIT University, Australia; ARC Industrial Transformation Research Hub for Intelligent Energy Efficiency in Future Protected Cropping (E2Crop), School of Science, RMIT University, Australia

Seawater, constituting >96% of Earth’s water resources, suffers from severe nitrate pollution due to excessive agricultural nutrients and poorly managed wastewater discharge [1,2]. Current remediation strategies (physicochemical and biological methods) face challenges such as high cost and limited applicability [3]. Recent advances in freshwater systems demonstrate the potential of converting nitrate into valuable ammonium (NH_4_^+^) via photo-/electrocatalysis. In contrast, piezocatalysis can directly harness ambient mechanical energy (e.g. sound, flow and vibration) to drive reactions without requiring light or electricity [4,5]. However, the application of piezocatalysis for nitrate reduction in seawater has not yet been reported.

**Figure 1.**
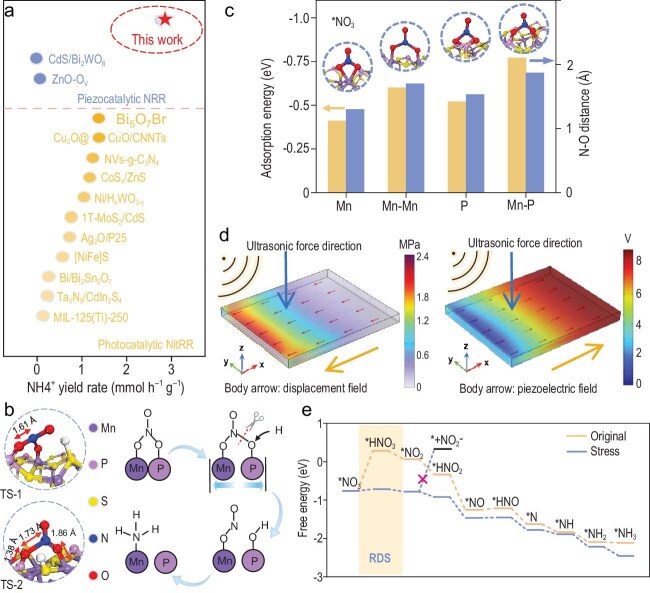
(a) NH_4_^+^ production performance evaluation of MnPS_3_ compared to current photocatalytic nitrate reduction reaction (NitRR) and piezocatalytic nitrogen reduction reaction (NRR). (b) Piezocatalytic NitRR mechanism on MnPS_3_ NSs. (c) Adsorption energies and corresponding N–O bond lengths for the four considered configurations on MnPS_3_ NSs. (d) Distribution of bulk strain and displacement fields in MnPS_3_ NSs under ultrasonic pressure. (e) Gibbs free energy profiles along the reaction pathway of piezocatalytic NitRR of MnPS_3_ NSs. Reproduced from Li *et al.* [6] with permission.

In a landmark study published in *National Science Review*, Professors Chao Liu and Chengzhong Yu’s group at East China Normal University found an elegant solution that simultaneously addresses these challenges: MnPS_3_ nanosheets (NSs) that harness naturally available mechanical energy to directly convert seawater nitrate into NH_4_^+^ [6]. The authors demonstrate that MnPS_3_ NSs function as highly efficient piezocatalysts, achieving an impressive 2.75 mmol h^−^^1^ g^−^^1^ NH_4_^+^ formation rate in simulated seawater under ultrasonication, in the absence of light, electricity or sacrificial agents, surpassing that of all previously reported analogous systems (Fig. 1a). This outstanding performance stems from a synergistic dual-site adsorption mechanism (Fig. 1b). *In situ* spectroscopy and density functional theory (DFT) calculations reveal that neighboring Mn and P atoms cooperatively bind NO_3_^−^ in a stable side-on configuration with an adsorption energy of −0.77 eV, thereby polarizing and

weakening the N–O bonds to facilitate reduction (Fig. 1c).

A central contribution of the study lies in revealing how mechanical deformation activates and enhances catalytic reactivity. External mechanical excitation induces lattice strain in MnPS_3_, producing a piezoelectric field that enhances charge separation and directs electrons to reactive Mn–P sites (Fig. 1d). DFT analysis indicates that strain narrows the bandgap by ∼0.57 eV and sharply lowers the free-energy barrier for the rate-determining hydrogenation step from +0.26 to −0.72 eV, rendering the transformation thermodynamically favorable. Meanwhile, sulfur sites on MnPS_3_ facilitate water dissociation, supplying active hydrogen while simultaneously suppressing the competing hydrogen evolution reaction (HER) owing to its high energy barrier (Fig. 1e).

Crucially, the system shows robust performance under real-world conditions. In a 2-L aquaculture seawater sample, vigorous stirring alone achieved 95.0% NO_3_^−^ conversion and 96.4% NH_4_^+^ selectivity within 120 min, while also exhibiting excellent durability over 100 cycles. The produced ammonia can be readily captured and crystallized into NH_4_Cl fertilizer, establishing an immediately applicable ‘waste-to-wealth’ pathway for coastal and aquaculture systems.

In summary, this work bridges nanoscale mechanisms with practical seawater applications, establishing MnPS_3_-based piezocatalysis as an energy-autonomous strategy for distributed nitrogen valorization [6]. The proposed principles of dual-site adsorption, strain-tunable electronic structure and multi-site hydrogen supply collectively offer a roadmap for the development of next-generation high-performance piezocatalysts. It is critical for facilitating efficient and challenging redox conversion, especially within the complex environment of seawater. Ultimately, this study highlights the transformative potential of harnessing ambient mechanical energy to drive selective chemical synthesis and close the nitrogen cycle in a sustainable, carbon-neutral manner.
